# RMOD: A Tool for Regulatory Motif Detection in Signaling Network

**DOI:** 10.1371/journal.pone.0068407

**Published:** 2013-07-12

**Authors:** Jinki Kim, Gwan-Su Yi

**Affiliations:** 1 Department of Information and Communications Engineering, Korea Advanced Institute of Science and Technology (KAIST), Daejeon, Republic of Korea; 2 Department of Bio and Brain Engineering, Korea Advanced Institute of Science and Technology (KAIST), Daejeon, Republic of Korea; Mount Sinai School of Medicine, United States of America

## Abstract

Regulatory motifs are patterns of activation and inhibition that appear repeatedly in various signaling networks and that show specific regulatory properties. However, the network structures of regulatory motifs are highly diverse and complex, rendering their identification difficult. Here, we present a RMOD, a web-based system for the identification of regulatory motifs and their properties in signaling networks. RMOD finds various network structures of regulatory motifs by compressing the signaling network and detecting the compressed forms of regulatory motifs. To apply it into a large-scale signaling network, it adopts a new subgraph search algorithm using a novel data structure called path-tree, which is a tree structure composed of isomorphic graphs of query regulatory motifs. This algorithm was evaluated using various sizes of signaling networks generated from the integration of various human signaling pathways and it showed that the speed and scalability of this algorithm outperforms those of other algorithms. RMOD includes interactive analysis and auxiliary tools that make it possible to manipulate the whole processes from building signaling network and query regulatory motifs to analyzing regulatory motifs with graphical illustration and summarized descriptions. As a result, RMOD provides an integrated view of the regulatory motifs and mechanism underlying their regulatory motif activities within the signaling network. RMOD is freely accessible online at the following URL: http://pks.kaist.ac.kr/rmod.

## Introduction

Regulatory motifs are patterns of activation and inhibition that appear repeatedly in various signaling networks and that show specific regulatory properties, such as oscillation, adaptaion and bistability [1]. For example, the interlinked positive and negative feedback loops controlling oscillations in the cell cycle and the circadian rhythm are *oscillatory motifs* [2], and the coupled positive feedback loops exhibiting bistable response in apoptosis and differentiation are *bistable switch motifs* [3]. The identification of regulatory motifs is important to understand the regulatory properties of signaling networks because these regulatory motifs have an essential role in exhibiting these regulatory properties and the regulatory properties of signaling networks are difficult to detect experimentally [4].

A small number of software tools were developed for statistical or numerical analysis of network topology to detect patterns that occur repeatedly within the network. For instance, the mFinder [5], MAVisto [6], FANMOD [7], SNAVI [8], and CytoKavosh [9] detect motifs that recur in the network much more often than expected in a random network; NetMatch [10] searches only for network motifs that match a user-defined query graph. In addition, the NetDS [11], a Cytoscape plugin, was recently developed to analyze a limited size of feedback and feedforward loops. However, it is difficult to identify regulatory motifs in the signaling networks using these software tools because these tools only focus on the statistical analysis of network topology without considering the diverse and complex network structures of regulatory motifs.

Recently, several computational modeling studies have revealed the minimal network structure of regulatory motifs for the representative bio-signaling such as oscillation, adaptation, and bistability and suggested them as the core regulatory mechanisms that control the cellular function of the biological system [3], [12], [13]. These regulatory motifs are all 2- and 3-node network topologies with signed directed edges, and they are parametrically robust in exhibiting dynamic behaviors. These studies assumes that the network structures of regulatory motifs often include various sizes of cascades composed of multiple molecules and their regulatory interactions with activation or inhibition and these cascades can be reduced into single regulatory interactions if we consider the effect of the cascade on the regulatory property. Thus, in order to detect the regulatory motifs, it is necessary to compress the signaling network into smaller network that retain the original network’s dynamic properties and analyze the compressed network using the compressed forms of regulatory motifs composed of 2- or 3-nodes.

Currently, there are several computational methods that involve simplifying complex networks [14], [15], [16]. These methods can be largely classified into two categories by focusing on network topological or dynamical properties. The methods focusing on network topological properties include coarse graining and filtering approach, which strive to preserve static topological properties, such as the small-world property, scale-freeness, fractality, or modularity [14], [15]. The other method focusing on network dynamic property is the kernel identification algorithm, which only provides the unique way to transform the original network into smaller network while preserving the network dynamic properties [16]. Since the kernel identification algorithm can be effectively applicable to the signaling network, it is possible to identify regulatory motifs and their regulatory properties using the compressed form of regulatory motifs after compressing the signaling network.

However, it is insufficient to detect regulatory motifs based on the network compression algorithm. Because the signaling network can have more than thousands of nodes and their regulatory interactions, it requires efficient subgraph search algorithm capable of detecting all occurrences of subgraphs matched with the compressed forms of regulatory motifs on large-scale signaling networks. Among the several subgraph search algorithms considering subgraph isomorphism [17], the VF2 algorithm is known as the most efficient method showing the less CPU times and memory consumption [18]. This algorithm extends a partial matching using a set of feasibility rules to decide whether to extend or backtrack and employs a depth-first search strategy in a recursive fashion. However, this algorithm is not effectively applicable to large-scale signaling networks because the depth-first search strategy causes exponential increases in search space as the size of network increases.

Here, we describe a RMOD, a web-based system for the analysis of regulatory motifs in the signaling network with a novel computational approach for identifying regulatory motifs and their properties. Considering that regulatory motifs can be reduced into 2 or 3-node networks and these networks have several isomorphic graphs, we developed efficient subgraph search algorithm using a novel data structure called path-tree, which is a tree structure composed of isomorphic graphs of regulatory motifs. We evaluated this algorithm using various sizes of signaling networks generated from the integration of various human signaling pathway resources and found that the speed and scalability of our algorithm outperforms those of other algorithm. By integrating this algorithm with network compression algorithm, we developed a RMOD, which is capable of identifying regulatory motifs after compressing the signaling network. RMOD includes interactive analysis and auxiliary tools that make it possible to manipulate the whole processes from building signaling network and query regulatory motifs to analyzing regulatory motifs with graphical illustration and summarized descriptions. RMOD can be freely accessible for non-commercial purposes at the following URL: http://pks.kaist.ac.kr/rmod.

## Materials and Methods

### Definitions

A graph or network consists of a finite set V of vertices and a finite set connecting edges E ⊆(V×V). A directed graph contains edge *e* = (*u*, *v*) ∈ E, which goes from vertex *u*, the source, to another vertex *v*, the target, Whereas an undirected graph has edges with no fixed orientation. The vertices u and v are *incident* with the edge e and *adjacent* to each other. Signed directed graph is a directed graph in which each edge has a positive or negative sign. A sub-graph of the graph G = (V, E) is a graph G_s_ = (V_s_, E_s_) where V_s_ and E_s_ ⊆ (V_s_×V_s_)∩E.

The *degree* of vertex is the total number of edges it is incident to. The *in-degree* and *out-degree* of a vertex is defined as the number of edges coming into the vertex and the number of edges going out of it, respectively. The *subgraph size* is defined in this paper as the number of vertices in the sub-graph.

Two sub-graph G_1_ = (V_1_, E_1_) and G_2_ = (V_2_, E_2_) are *isomorphic* if there is a one-to-one correspondence between their vertices, and there is an edge directed from one vertex to another vertex of one subgraph if and only if there is an edge with the same direction between the corresponding vertices in the other subgraph. The problem of finding an isomorphic subgraph is believed to be a problem for which no efficient solution exists, i.e., it belongs to the class of NP-complete problems.

For a particular sub-graph G_p_, all subgraphs isomorphic to G_p_ in the network are considered as *matches* of G_p_. Network motifs are defined as subgraphs, which have higher occurrences of *matches* in the network than in random networks of equal size. Regulatory motifs are subgraphs of signed directed graph that appear repeatedly in various signaling networks and show specific regulatory properties such as oscillation, adaptaion and bistability. The compressed forms of regulatory motifs in this paper are defined as regulatory motifs with minimal nodes.

### Known Regulatory Motifs

**Figure 1:**
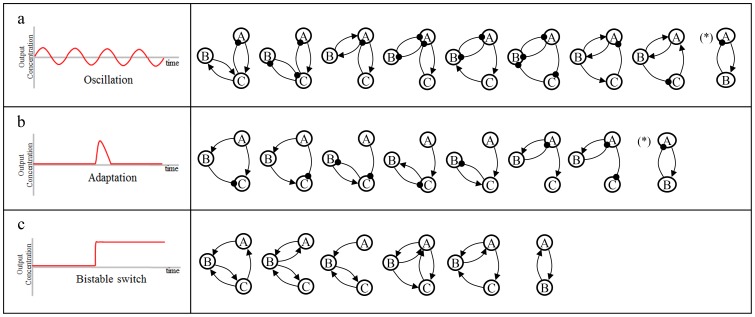
Known regulatory motifs in non-isomorphic relationship. (a) Oscillation motif (b) Adaptation motif (c) Bistable switch motif. A, B, C in the circle represent enzymes that catalyze reaction in their active state, For example, A → B indicates that A converts B from its inactive state to active state and A ⊣ B indicates that A convert B from its active state to inactive state. * means that the network size should be more than equal to three for exhibiting dynamic behaviour.

We collected regulatory motifs for representative bio-signaling such as oscillation, adaptation, and bistability from individual literatures. These are 12 oscillatory motifs [12], 11 adaptation motifs [13] and 12 bistable switch motifs [3], which were identified from an individual study based on mathematical modeling and simulation (See File S1). These regulatory motifs are all 2- and 3-node network topologies with signed directed edges, and they are parametrically robust in exhibiting dynamic behaviors. Since some of these regulatory motifs are in isomorphic relationship or can be compressed, we converted them into compressed forms of non-isomorphic networks as shown in Figure 1.

### Regulatory Motif Identification

**Figure 2:**
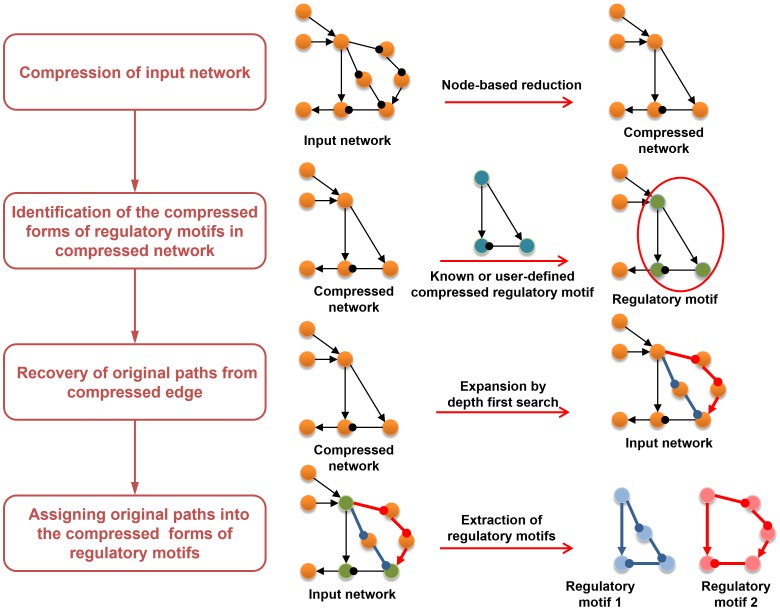
Overview of regulatory motif identification process.

Figure 2 presents a schematic diagram depicting the individual steps for regulatory motif identification. The details of the each step are described below.

#### Step 1

The input network is compressed based on the regulatory relationship between neighboring edges. We used the node-based reduction part of a kernel identification algorithm, which replaces the neighborhood subnetwork of each node with a smaller one without disrupting the network dynamics [16]. (See File S2).

#### Step 2

Using our subgraph search algorithm, we search the compressed network for the subgraphs matched with the compressed forms of regulatory motifs. This will be discussed in detail later.

#### Step 3

The original paths from the compressed edge of matched subgraphs are recovered from the input network. The compressed edges are expanded by using a depth-first search with two constraints. The first constraint is that the path includes only eliminated nodes, except the start and end nodes. The second constraint is that total regulatory effect of the path is the same as that of the compressed edge.

#### Step 4

The matched subgraphs and original path information are integrated into the input network and then, the individual regulatory motifs are extracted by selecting one original path from each edge of the matched subgraphs.

### Subgraph Search Algorithm

The searching (matching) process between a query regulatory motif and a given input network consists in the determination of mapping which associates nodes of the query regulatory motif to nodes of the input network. The solution to the searching problem could be obtained by computing all the possible partial mapping and selecting the ones satisfying the wanted mapping type. In order to reduce the number of paths to be explored during the search, our algorithm uses a novel data structure called a path-tree as feasibility rules for partial mapping and employs the ESU algorithm as a search strategy [19].

**Figure 3:**
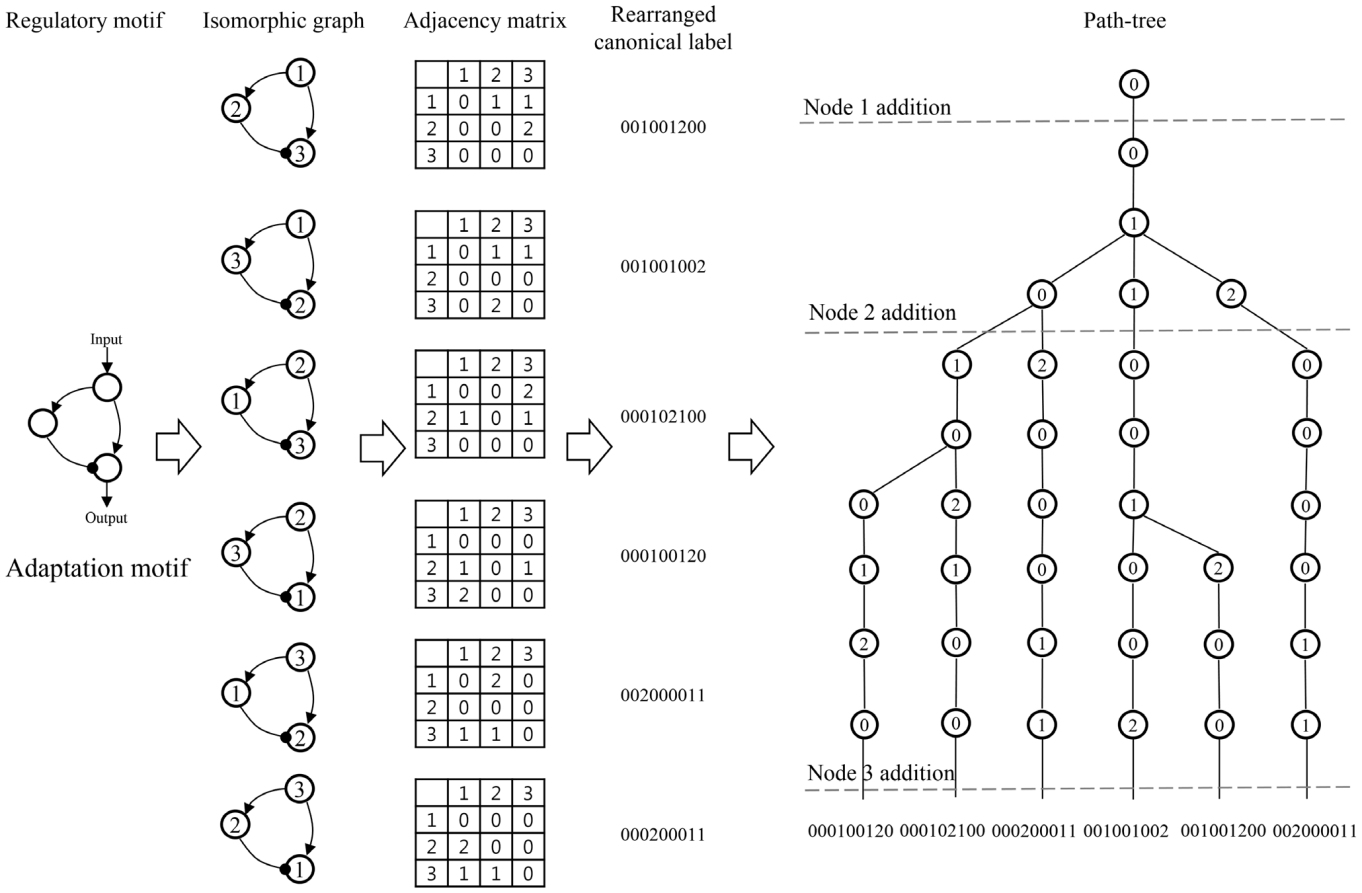
The construction of a path-tree for the adaptation motif as an example.

The path-tree is a novel data structure to evaluate the feasibility of adding newly explored node into the partial mapping. It is composed of all isomorphic graphs of query regulatory motifs and organized into a tree structure to directly evaluate the newly created edges. Figure 3 illustrates the construction of a path-tree for the adaptation motif as an example. It is constructed by loading canonical labels, which are the rearranged elements of adjacency matrices of isomorphic graphs. The isomorphic graphs of regulatory motifs are generated by the canonical graph labeling algorithm NAUTY [20] and the canonical labels are made by selecting and concatenating diagonal, row and column elements. For example, the elements in the 3×3 adjacency matrix are selected in the following order: (1,1), (2,2), (2,1), (1,2), (3,3), (3,1), (3,2), (1,3), and (2,3).

**Figure 4:**
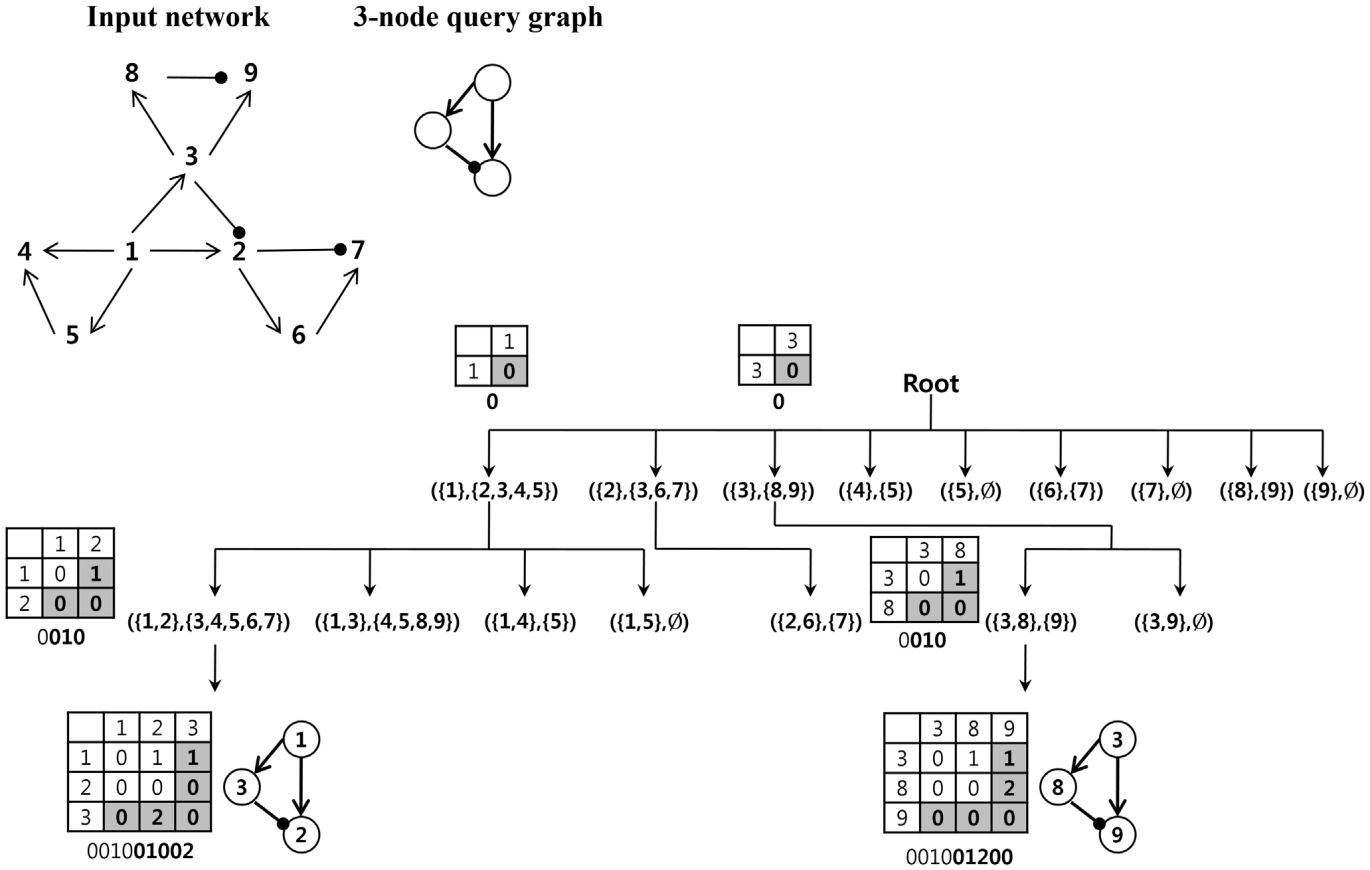
The process of searching for adaptation motif in the input network as an example.

The ESU algorithm is employed to efficiently explore the search space. Although the ESU algorithm was originally developed for efficiently enumerating all *k*-node subgraphs, it can be effectively used to guide the paths to be explored during the search. The ESU algorithm first assigns an integer label on each node in the input network and finds all *k*-node subgraphs that a particular node participated in, then removes that node and subsequently repeats the process for the remaining nodes. During this process, it enumerates all *k*-node subgraphs exactly once. This enumeration process is directly applied to explore the path to extend a partial mapping. Figure 4 illustrates the process of searching for adaptation motif in the input network. It is assumed that the path-tree for the adaptation motif is already loaded in the memory. Our algorithm explores the input network node based on both the integer label and connectivity and extends a partial mapping using a path-tree to decide whether to extend or backtrack. It prints the subgraph covering all the partial mapping when a partial mapping reaches the end of the path-tree. (See File S3.).

From the searching process, we can approximately estimate the time complexity of searching for all occurrences of *k*-node subgraph. If we suppose that the input network is fully connected graph with *N* nodes and the query regulatory motif is *k*-node graph, the total number of comparison is 
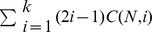
 (C(n, k) is the number of different combinations of k elements through n elements) because the total number of explored nodes is 
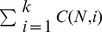
 and the number of increased edges from *k*−1-node to *k*-node graph is *2k−1*. Since it is difficult to calculate the equation, we approximate the equation by changing *k*-node graph into *N*-node graph as the upper bound: 
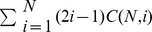
. Hence, the total number of comparison is *2^N^(N−1)*, and the time complexity is approximately *O*(*N2^N^*). The size of subgraph is practically less than N, and the most of the explored paths are pruned; therefore, the algorithm runs several orders of magnitude faster.

### Biological Network Dataset

To test the speed and scalability of our subgraph search algorithm, we used different sizes of signaling networks obtained from the integration of human signaling pathways. To build up the integrated signaling network, we collected the signaling molecules (most of them are proteins) and the activation or inhibition interactions between these molecules from the widely used pathway databases, Kyoto Encyclopedia of Genes and Genomes (KEGG) [21], NCI/Nature Pathway Interaction Database (PID) [22], BioCarta [23], Reactome [24], and PharmGKB [25]. As genes and proteins often have multiple synonyms, we used the Entrez GeneID for genes and their products as a cross-reference for ID mapping. We also excluded the inconsistent interactions with both activation and inhibition from the integrated signaling network. As a result, we obtained the integrated signaling network containing 9649 nodes and 22347 edges that include 18398 activation edges (positive edges) and 3949 inhibitory edges (negative edges). Finally, we generated 6 different sizes of signaling networks by selecting the signaling pathways as shown in Table 1. (See File S4).

**Table 1:** Signaling networks generated from the integration of signaling pathways.

Network name	Number of pathways	Number of nodes	Number of edges
SN1	1	68	89
SN2	2	137	169
SN3	14	510	1356
SN4	20	1052	2364
SN5	150	2746	10959
SN6	1153	9649	22347

### System Architecture

**Figure 5:**
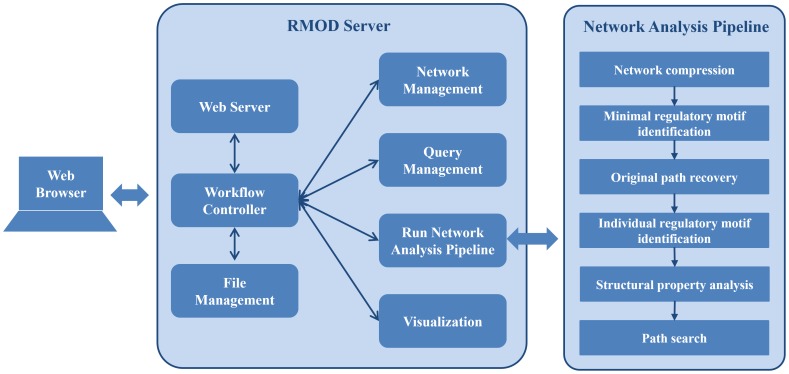
RMOD system architecture. The Web server hosts the RMOD Web application and accepts user requests via standard Web browsers. The RMOD server handles user requests for network and query management, run the network analysis pipeline and presents the result of analyzed input via network viewer. The file management module stores all network, regulatory motif and job-related data.

Figure 5 shows the system architecture of RMOD. It comprises of a Web application server (RMOD server) that provides an easy-to-use graphical user interfaces (GUIs) accessible via a Web browser, an embedded file management module for storing network, regulatory motif and other job-related data, and network analysis pipeline for regulatory motif identification. The RMOD server and network analysis pipeline can be accessed by multiple users through the RMOD server GUI using standard Web browsers. The RMOD GUI provides four main functions to the users: (i) network management for uploading and manipulating input network; (ii) query management for manipulating and generating query regulatory motif; (iii) job submission for running the network analysis pipeline; (iv) graphical visualization of the analyzed input network.

The network analysis pipeline is a standalone application that is initiated by the RMOD server at the user’s request and run in batch mode. As shown in Figure 3, the network analysis pipeline includes regulatory motif identification module and additional analysis modules for characterizing structural features of detected regulatory motif. The structural property analysis module calculates the sign, the length of the path and the size of individual regulatory motifs. The path search module, which is used for searching regulatory motif in user-defined path, finds the shortest paths that connect two nodes in network using Floyd algorithm [26] and selects regulatory motifs included in user-defined path.

The RMOD system has been designed for easy integration with future network analysis module. The RMOD server was developed using Perl on a Linux platform with an Apache web-server. The Web interface was designed and implemented using Perl, HTML and Asynchronous Javascript and XML (AJAX). Cytoscape Web was also used in the Web interface for network and regulatory motif visualization [27]. AJAX was adopted for controlling the execution of various functions of Cytoscape Web and making Web pages more interactive without page reloading. The core functionalities in the network analysis pipeline were implemented using C++ programming language and standard template library.

**Availability and Requirements**

**Project name:** RMOD

**Project home page:** http://pks.kaist.ac.kr/rmod

**Operating system:** Linux

## Experiments

Among the several steps in identifying regulatory motifs, the search for subgraphs in the second step is a key factor in determining the performance of regulatory motif identification. In this section, we report the experimental results obtained from testing our subgraph search algorithm and the VF2 algorithm [18]. We chose to compare with the VF2 algorithm, because it is the most efficient sub-graph isomorphism algorithm based on time [17].

### Experimental Setup

The computer system used in these experiments was equipped with 3.4 GHz Intel Core i7 processor (4 cores) with 4 GB RAM running Cent OS Linux 5.5. All implementations for these experiments were written in C++. The VF2 algorithm was the optimized versions as presented in the VFLib library.

### Accuracy

We evaluated the accuracy of our subgraph search algorithm by comparing the number of detected subgraphs between our algorithm and the VF2 algorithm. All graphs with size 3–5 nodes were generated from signaling network SN1 and SN2 by using the FANMOD and classified into non-isomorphic-graphs. Both algorithms were tested on the signaling networks SN1 and SN2 with non-isomorphic-graphs. The result shows that our algorithm could successfully detect all subgraphs in each signaling network as the VF2 algorithm could. (data not shown).

### Scalability

Since all the subgraphs in our test datasets were correctly identified by our algorithm, we attempted to test the speed and scalability of our algorithm with our signaling network datasets. We measured the average run-time for all occurrences of subgraph using 50 *k*-node query graphs (3≤k≤6), which are randomly selected non-isomorphic subgraphs generated by the FANMOD, and compared the performance of our algorithm with that of the VF2 algorithm. If the number of non-isomorphic subgraphs in signaling networks is less than 50, all non-isomorphic subgraphs in the signaling network were used as query graphs.

**Figure 6:**
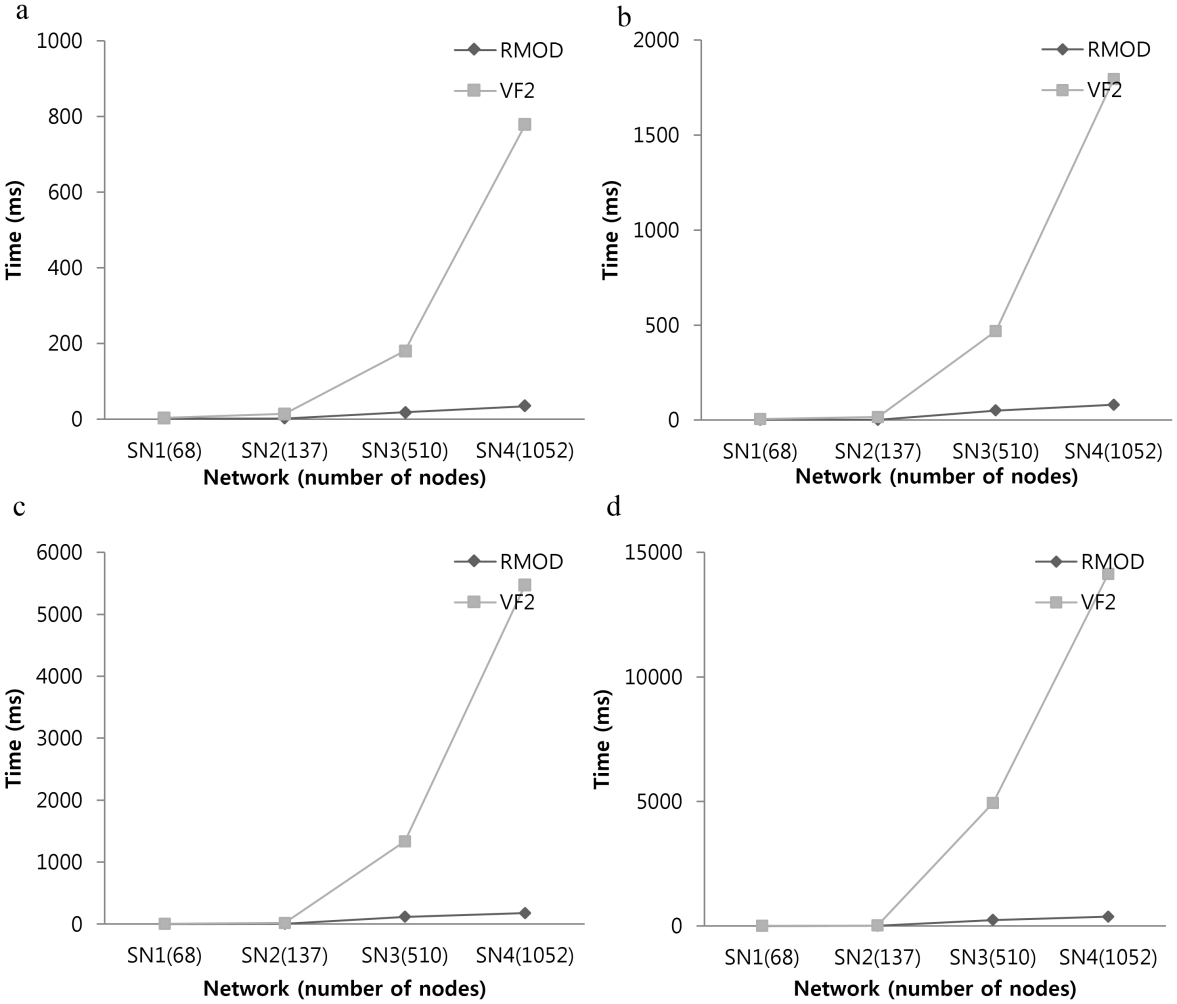
The run-time comparisons between the RMOD and the VF2 algorithm. The average run-times of searching for all occurrences of a subgraph were measured against various signaling networks. Illustrated results are for (a) 3-node subgraph search (b) 4-node subgraph search (c) 5-node subgraph search (d) 6-node subgraph search. Times are given in milliseconds (ms).

Figure 6 shows the average run-time of searching for all occurrences of a subgraph in various sizes of signaling networks, where the size of a single query graph varies. We see that the run-time of our algorithm approximately increases in linear as the size of network increases. We also see that our algorithm shows a significantly smaller run-time than that of the VF2 algorithm, and the difference between our algorithm and the VF2 algorithm becomes even more prominent when the network is large. For example, our algorithm shows about 376 milliseconds (ms) in average run-time for detecting 6-node sub-graphs in signaling network SN4 whereas the VF2 algorithm shows about 14128 ms. This difference results from the exponential increase in the path to be explored in the VF2 algorithm. Table 2 shows the experimental results obtained from testing our algorithm on signaling network SN5 and SN6. Because of the high growth of the number of subgraphs, these large networks were tested for subgraphs of size 3, 4, and 5. The VF2 algorithm is not tested because it requires a large amount of times to detect subgraphs on the signaling network SN5 and SN6 and it shows memory overflow on signaling network with more than 8000 nodes for detecting 3-node subgraphs (data not shown). Since the speed of our algorithm was obtained from real signaling networks, we consider the run-time of detecting regulatory motifs against the any sizes of signaling networks can be estimated by interpolating or extrapolating our experimental results.

**Table 2:** Computational cost for RMOD algorithm on large signaling networks.

	Query graph size		
Network	3	4	5
SN5	2545.91	51137.15	446923.56
SN6	4223.84	64478.95	640834.4

Rows indicate the running time (milliseconds) of our subgraph search algorithm for each query graph size.

## Results

To illustrate the effectiveness of RMOD in signaling network, we explain the functionalities of interactive analysis and the auxiliary tools that make it possible to identify regulatory motifs and analyze their structural properties. Then we show each step of the regulatory motif analysis, staring from the network creation and ending with the visualization of the analyzed network with an example of apoptosis regulation network [28]. This example is also available for review on the RMOD web page: http://pks.kaist.ac.kr/rmod.

### Creating a Network

**Figure 7:**
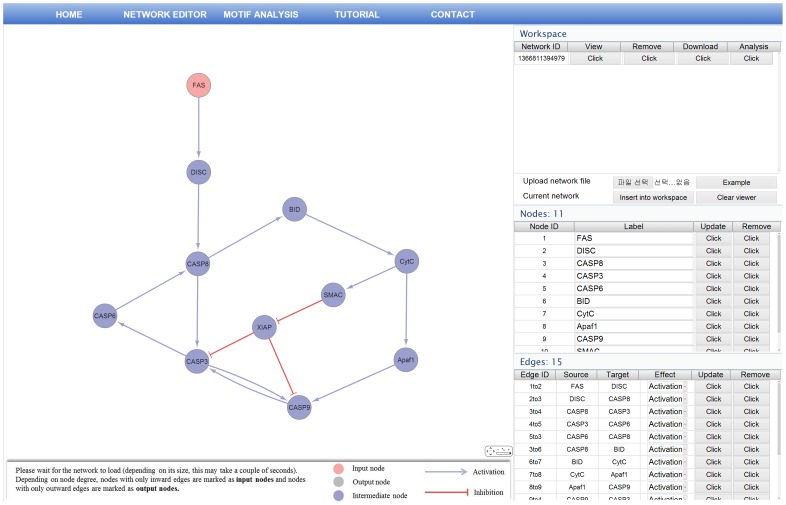
The network editor interface. The network editor allows users to create or edit input network.

The first step in analyzing regulatory motif is to create a regulatory network where the edge represents the regulatory effect, such as activation and inhibition. RMOD allows users to create regulatory networks either by uploading the input data file or by selecting and editing preloaded network files in the network editor interface. It accepts the input data files in a format where the first, second, third columns denote regulator, relation, and target, respectively. The regulator and the target can be any string label, but the relation contains only of two predefined characters, such as + and −, which correspond to activation and inhibition, respectively. Users can also modify and update any selected network via network viewer or data tables by adding or deleting the nodes and edges in the network. In Figure 7, we show the network editor interface with an example of uploaded apoptosis regulation network with 11 nodes and 15 edges. As shown in Figure 7, the network elements in apoptosis regulation network show various color and shape in the network editor interface. This is because the RMOD represents the structural properties with different color and shape. The nodes with only inward edges are marked as input nodes, and the ones with only outward edges are marked as output nodes. The edges also have different color and shape depending on their properties, such as activation and inhibition.

### Generating Query Regulatory Motifs

**Figure 8:**
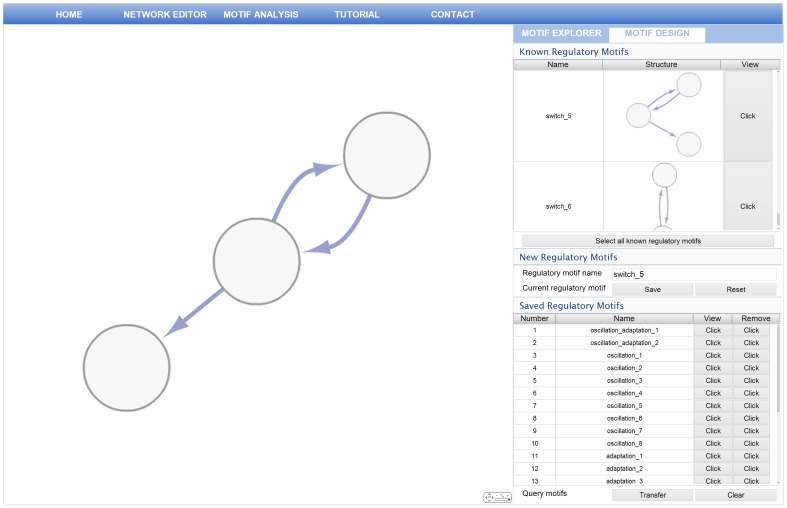
The motif designer interface. The motif designer enables users to select or create query regulatory motifs.

In order to identify regulatory motifs against the input network, it is necessary to define query regulatory motifs, which are compressed forms of regulatory networks representing specific regulatory properties. Currently, since small numbers of regulatory motifs were identified by using mathematical modeling and simulation, RMOD provides flexible methods for generating query regulatory motifs via the motif designer interface. RMOD allows users to select known regulatory motifs or edit the nodes and edges to build novel regulatory motifs in the motif designer interface. As described in Materials and methods section, the available regulatory motifs are bistable switches, oscillation motif and adaptation motif. In Figure 8, the motif designer interface shows one of bistable switch motifs selected in known regulatory motif list. After creating regulatory motif, users should save the regulatory motif in the motif designer interface. When users click the save button, it automatically checks isomorphic relationship by comparing saved regulatory motifs with currently selected regulatory motif and saves it into saved regulatory motif list. These saved regulatory motifs are finally transferred into the network analysis pipeline as query regulatory motifs.

### Analyzing a Network

After creating the input network and query regulatory motifs, RMOD provides two options for analyzing input network: (i) analysis of the whole network or (ii) analysis of nodes along a user-defined path. To designate user-defined path, the users can query a network by selecting a source node and a target node and then RMOD finds the shortest paths that connect these 2 elements in network. After analyzing input network, RMOD shows the summarized result of regulatory motif analysis.

**Figure 9:**
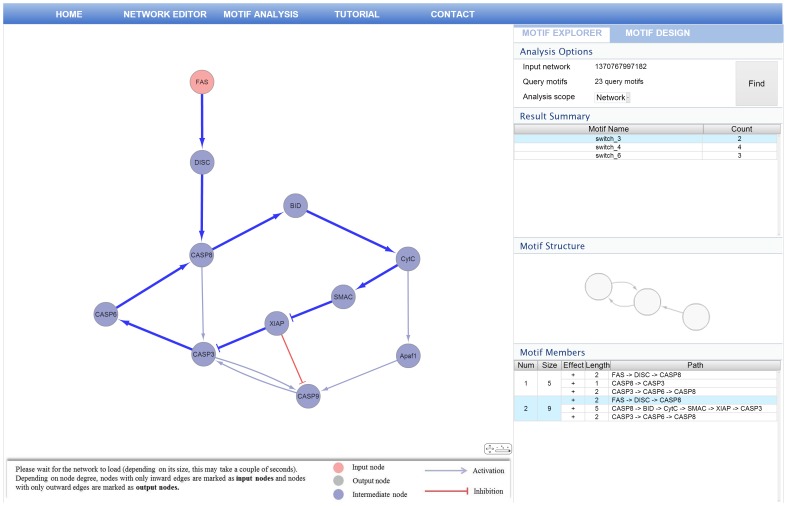
The motif explorer interface. The motif explorer allows users to analyze the input network and show the result of regulatory motif analysis.

As shown in Figure 9, it allows users to examine how often detected regulatory motif match the query regulatory motifs. When users select a regulatory motif, it shows the compressed forms of regulatory motifs in the motif structure viewer and provides the individual regulatory motif information from the motif member table. The individual regulatory information includes a list of motif member nodes and structural properties, such as path, sign, length between nodes, and the size of regulatory motif. To facilitate the visual exploration of the occurrence of a regulatory motif within the analyzed network, it allows users to highlight the matches by clicking the individual regulatory motif in motif member table. In Figure 9, the motif explorer interface shows the highlight of bistable switch motif in the network viewer and the motif member table.

### Example of Regulatory Motif Analysis

The hallmark of apoptosis is the all-or-none activation of caspase-3 in response to various apoptotic signals [29]; therefore, we used the apoptosis regulation network composed of 11 nodes and 15 edges. We used RMOD to examine the regulatory motifs obtained from the analysis of the apoptosis regulation network.

As shown in Figure 9, our analysis result shows that the apoptosis regulation network contains 9 bistable switch motifs. Interestingly, these are all classified into 3 types of bistable switch motifs, including positive feedback loop. Their individual regulatory motifs show different sizes, even though individual regulatory motifs are included in the same compressed forms of regulatory motif class. Remarkably, it shows that caspase-3 is involved in all positive feedback loops in detected bistable switch motifs. Therefore, we investigated several computational modeling studies focusing on the bistable activity of caspase-3 in apoptosis. We found a positive feedback loop including caspase-3, which is necessary to cause bistable response of caspase-3 [28], [30]. Our analysis result implies that the bistable behaviour of caspase-3 in an apoptosis regulation network can occur through various bistable-switch motifs, depending on the cellular conditions.

## Discussion

We have developed a novel computational approach designed to identify regulatory motifs and their properties in a signaling network. It is necessary to understand the regulatory mechanisms and their dynamic regulatory properties to get insight into cellular functions. However, it is still difficult to detect dynamic regulatory properties of specific signaling network using experimental techniques because it requires measurements with high temporal and spatial resolution [4]. We thus tried to solve this issue by developing a novel computational method to detect potential regulatory motifs capable of exhibiting dynamic regulatory properties. Our approach accomplishes the identification of the regulatory motifs by compressing the signaling network and detecting the compressed forms of the regulatory motifs by using our subgraph search algorithm to finds various network structures of the regulatory motifs.

Our subgraph search algorithm enabled us to efficiently detect the regulatory motifs in large-scale signaling networks. It employs the ESU algorithm to reduce the search space and uses a path-tree to evaluate the feasibility of newly added node. However, it has a limitation that the construction of the path-tree totally depends on the isomorphic graphs of query regulatory motifs. The generation of isomorphic graphs requires a large amounts of times when the size of regulatory motif is larger than 5. In order to overcome this limitation, RMOD system provides a path-tree library for known regulatory motifs and allows reusing the library even if users make new regulatory motif using motif design tool. We consider continuously updating our path-tree library, whenever a new regulatory motif is discovered.

Introducing network compression into regulatory motif detection enables us not only to detect regulatory motifs with various network structures, but also to reduce network complexity by replacing original network into smaller network. However, it does not always guarantee the run-time reduction. Actually, even though the network compression of known human signaling network, which is composed of 1240 nodes and 3144 edges, shows 23% node reduction, the average run-time of detecting 6-node subgraphs increases [31], whereas the average run-times of detecting subgraphs with size 3–5 nodes decrease. This is the reason why the effect of subgraph creation on the run-time is larger than that of reduction in network size (data not shown).

In summary, RMOD is a web-based system for the analysis of regulatory motifs in a signaling network with a novel computational approach for identifying regulatory motifs and their properties. It includes interactive analysis and auxiliary tools that make it possible to manipulate the whole processes from building signaling network and query regulatory motifs to analyzing regulatory motifs with graphical illustration and summarized descriptions. Therefore, it can be used both as a discovery tool for analyzing regulatory motifs to understand cellular function in natural systems and as a design tool for identifying specific circuits, which can be used to engineer synthetic circuits. In the near future, RMOD will be extended to have more powerful functions by integrating a simulation tools.

## Supporting Information

File S1 **List of original known regulatory motifs for (a) oscillation** [**12**] **, (b) adaptation** [**13**] **and (c) bistable switch** [**3**] **.** A, B, C in the circle represent enzymes that catalyze reaction in their active state, For example, A → B indicates that A converts B from its inactive state to active state and A ⊣ B indicates that A convert B from its active state to inactive state. The input is applied to species A and the output is taken to be the concentration of the active forms of C. * means that the network size should be more than equal to three for exhibiting dynamic behavior.
(DOCX)

File S2 **The flow diagram illustrating network compression.** This flow diagram is the node-based reduction part of a kernel identification algorithm [16]. Signaling network can be represented by a signed graph G = (V, E), where V is a set of nodes and E is a set of edges with signs. Each edge can be represented by e_ij_ = (v_i_, v_j_, σ_ij_), where v_i_ is a start node, v_j_ is an end node, and σ_ij_ is a sign (+1, 0, or −1) of the edge. σ_ij_ = 0 denotes that two nodes v_i_ and v_j_ are not connected by an edge.
(DOCX)

File S3 **Pseudo-codes for subgraph search algorithm.**
(DOCX)

File S4 **Provides the signaling networks generated from the integration of signaling pathways.**
(XLSX)
