# Supplementary figures and images for: RMOD: A Tool for Regulatory Motif Detection in Signaling Network
Source: PLoS One. 2013 Jul 12;8(7):e68407. doi: 10.1371/journal.pone.0068407 (PMC3710000; doi:10.1371/journal.pone.0068407)

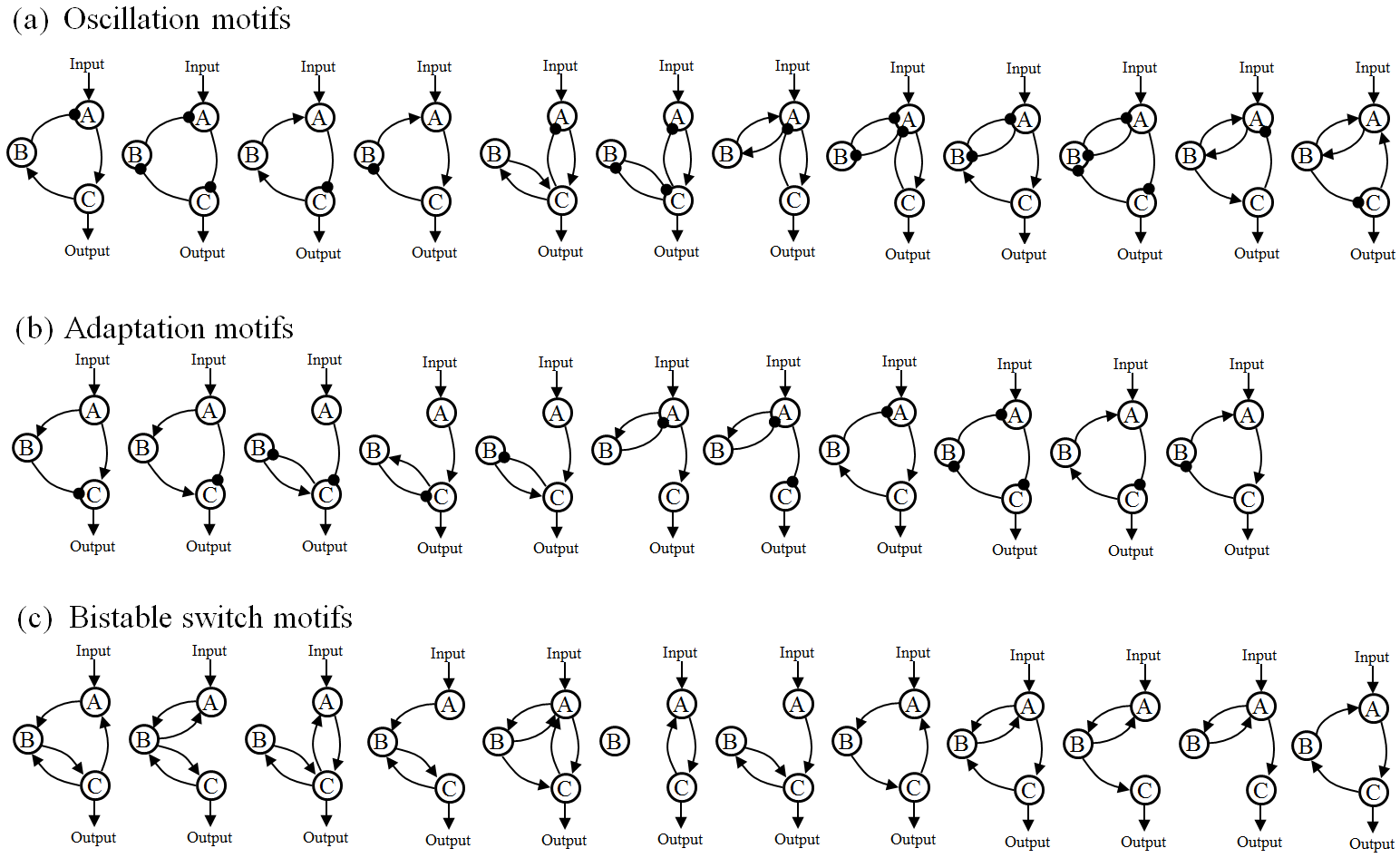

Supplement: File S1 — List of original known regulatory motifs for (a) oscillation [12] , (b) adaptation [13] and (c) bistable switch [3] . A, B, C in the circle represent enzymes that catalyze reaction in their active state, For example, A → B indicates that A converts B from its inactive state to active state and A ⊣ B indicates that A convert B from its active state to inactive state. The input is applied to species A and the output is taken to be the concentration of the active forms of C. * means that the network size should be more than equal to three for exhibiting dynamic behavior. (DOCX) [file pone.0068407.s001.docx]

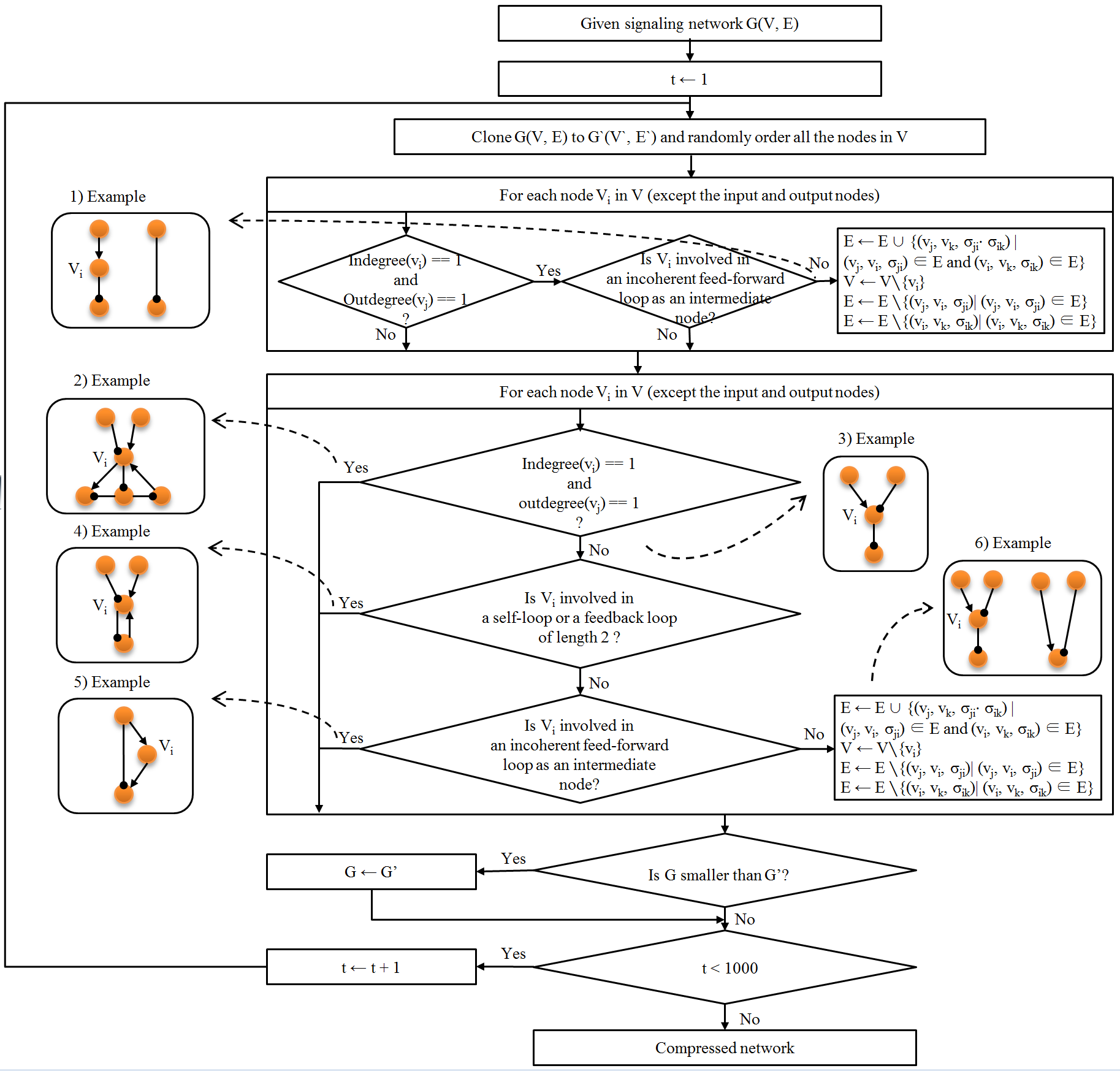

Supplement: File S2 — The flow diagram illustrating network compression. This flow diagram is the node-based reduction part of a kernel identification algorithm [16]. Signaling network can be represented by a signed graph G = (V, E), where V is a set of nodes and E is a set of edges with signs. Each edge can be represented by eij = (vi, vj, σij), where vi is a start node, vj is an end node, and σij is a sign (+1, 0, or −1) of the edge. σij = 0 denotes that two nodes vi and vj are not connected by an edge. (DOCX) [file pone.0068407.s002.docx]
