# Supplementary material for: RMOD: A Tool for Regulatory Motif Detection in Signaling Network
Source: PLoS One. 2013 Jul 12;8(7):e68407. doi: 10.1371/journal.pone.0068407 (PMC3710000; doi:10.1371/journal.pone.0068407)
Supplement: File S3 — Pseudo-codes for subgraph search algorithm. (DOCX) [file pone.0068407.s003.docx]

**Pseudo-code for sub-graph search algorithm**
